# Mortality and low C-peptide levels in children and young people diagnosed with type 1 diabetes in Cameroon: a case series from the young-onset diabetes in sub-Saharan Africa study

**DOI:** 10.11604/pamj.2025.50.62.46186

**Published:** 2025-02-26

**Authors:** Jean Claude Katte, Emmanuel Gwan, Christiale Kenne Feuatsap, Liliane Kamdem, Adele Chetcha, Mesmin Yefou Dehayem

**Affiliations:** 1 Department of Clinical and Biomedical Sciences, University of Exeter Medical School, Exeter, United Kingdom; 2 National Obesity Centre and The Endocrinology and Metabolic Diseases Unit, Yaoundé Central Hospital, Yaoundé, Cameroon; 3 Department of Non-Communicable Diseases, RSD Institute, Yaoundé, Cameroon; 4 Faculty of Medicine and Biomedical Sciences, Department of Internal Medicine and Specialities, University of Yaoundé I, Yaoundé, Cameroon

**Keywords:** Type 1 diabetes, mortality, C-peptide levels, children and young people, Cameroon, sub-Saharan Africa

## Abstract

Type 1 diabetes-associated mortality in Africa remains high despite several coordinated international initiatives. Therefore, understanding the causes of death will guide context-specific public health interventions. This descriptive case series examined the number and causes of death among children and young people (CYP) with type 1 diabetes (T1D) in the young-onset diabetes in sub-Saharan Africa (YODA) study in Cameroon. Among 313 CYP recruited between 2019 and 2022, 23 deaths were recorded (56.5% male). At recruitment, the median (IQR) age at diagnosis was 16 (13-18) years, BMI 21.5 (18.9-24.7) kg/m^2^, and plasma C-peptide concentration 116 (37-210) pmol/L, respectively. Causes of death included hypoglycaemia (7 cases), diabetic ketoacidosis (5 cases), infections (4 cases), undefined causes (4 cases), chronic liver disease (2 cases), and chronic kidney disease (1 case). Most deaths (17/23, 73.9%) occurred in individuals with significantly low plasma C-peptide levels (<200 pmol/L), signifying severe insulin deficiency. Hypoglycaemia deaths (85.7%, 6/7) were also common at significantly low C-peptide levels (<200 pmol/L). Nearly half (11/23, 47.8%) of the deaths occurred outside the hospital setting. These findings describe a possible relationship between low C-peptide and mortality for CYP living with T1D in Cameroon which should be investigated thoroughly prospectively.

## Introduction

Type 1 diabetes (T1D) is a chronic metabolic condition characterised by absolute or near-absolute insulin deficiency due to the destruction of the insulin-secreting beta cells of the pancreatic islet. It is one of the most common diseases of children and young people (CYP), although it can affect older and older people [1]. The increasing burden of T1D is a primary healthcare concern worldwide. Globally, it affects about 8.4 million individuals, with about 1.5 million CYP below 20 years of age [2,3]. The incidence of T1D varies geographically, with higher rates observed in Europe and North America compared to sub-Saharan Africa (SSA) [4]. Despite the lower incidence and prevalence of T1D in SSA compared to other regions worldwide, recent studies have suggested a continuous rise in T1D cases [5]. This rise in the number of T1D cases also coincides with a disproportionately high mortality rate compared to that seen in high-income countries [6].

Classically, T1D-associated mortality in SSA has been attributed mainly to poor insulin access, and within the last two decades, several initiatives have been set up to address this challenge. The Changing Diabetes in Children (CDiC) programme, one of such free insulin-providing initiatives, has now been implemented in 30 lower-and-middle-income countries (LMICs), including 17 countries in SSA [7]. Many clinical and anecdotal reports have documented the benefits and impact of these initiatives in improving survival and overall quality of life of CYP with diabetes in the African continent [8-10]. However, despite these efforts, much is still to be achieved to further reduce T1D-related mortality in SSA to the level observed in high-income countries [1]. Therefore, studies examining the causes and determinants of mortality in CYP with T1D are still relevant and will be critical to inform the effective development of interventions. In this descriptive case series, we aimed to establish the number and causes of mortality within a cohort of carefully selected and phenotyped CYP diagnosed with T1D recruited into the Young-Onset Diabetes in sub-Saharan Africa (YODA) study in Cameroon.

## Methods

**Study design and data collection:** we conducted a descriptive review of death cases using data from the YODA study in Cameroon. The YODA study recruited CYP diagnosed with T1D at an age below 30 years, receiving insulin treatment at the time of recruitment between 2019 and 2022 (ClinicalTrials.gov identifier: NCT05013346). We reviewed all registered death cases among the YODA study participants in Cameroon and extracted relevant data into a structured data collection form. We recorded their sociodemographic (age at diabetes diagnosis, insulin regimen and dose), anthropometric (weight, BMI), and biological (HbA1c, plasma C-peptide) data collected at the time of recruitment, and data in their medical records on the circumstances of death (causes and place of death). To ensure the accurate recording of the cause-of-death information, we also verified the paper-back clinic medical records of all the death cases.

**Data management and analysis:** all collected data was entered into a Microsoft Excel 2013 spreadsheet and was checked item-by-item by a separate research staff for accuracy and consistency. Descriptive analysis was used to summarise participants' demographics, C-peptide levels, treatment details, and causes of death using the IBM SPSS Statistics version 23.0 (Chicago, Illinois, USA). Data were reported as median (interquartile range) for continuous variables and frequency (percentage) for categorical variables.

**Ethical considerations:** ethical approval was obtained from the Cameroon National Ethical Review Board for Human Health for the YODA study, and administrative authorisation was obtained from the central coordination unit of the CDiC programme in Cameroon. We anonymised all data to ensure participant confidentiality. This study adhered to the ethical principles outlined in the Helsinki Declaration [11].

## Results

**General characteristics of the death cases:** among the 313 individuals enrolled in the YODA study in Cameroon, we recorded 23 deaths after 53 months, consisting of 13 males (56.5%). The median (IQR) time from recruitment to the death recording was 20 (8-30) months. The median (IQR) age at diagnosis was 16 (13-18) years, ranging from 8-27 years, and the median BMI was 21.5 (18.9-24.7) kg/m^2^. The median (IQR) HbA1c and C-peptide levels were 94 (69-104) mmol/mol and 116 (37-210) pmol/L, respectively. Table 1 shows the individual clinical characteristics of all the death cases.

**Table 1:** clinical characteristics of the 23 death cases

Cases	Age at diabetes diagnosis	Age at recruitment	Period in YODA (Days)	Received free insulin (Y/N)	Total insulin dose (IU/day)	Weight (Kg)	Plasma C-peptide (pmol/L)	Cause of death	Place of death
P1	19	27	5	N	30	53	120	Hypoglycaemia	Out Hospital
P2	18	22	46	Y	40	79	152	DKA	Hospital
P3	27	29	48	N	88	47	11	Undefined	Out Hospital
P5	11	24	101	Y	54	82	275	Infections	Hospital
P6	22	32	155	N	62	67	3	Infections	Hospital
P7	16	17	235	Y	42	47	33	Hypoglycaemia	Out Hospital
P8	13	19	262	Y	50	38	3	Hypoglycaemia	Out Hospital
P8	22	31	371	N	40	91	618	Undefined	Hospital
P9	14	22	390	Y	114	69	41	Hypoglycaemia	Out Hospital
P10	9	20	405	Y	86	52	3	Infections	Out Hospital
P11	16	20	406	Y	82	55	116	CLD	Unknown
P12	14	23	602	N	180	47	89	Infections	Out Hospital
P13	14	14	694	Y	56	56	174	Hypoglycaemia	Out Hospital
P14	16	17	707	Y	70	45	124	DKA	Hospital
P15	18	26	900	Y	42	62	41	Hypoglycaemia	Hospital
P16	13	14	1021	Y	48	46	198	DKA	Hospital
P17	11	13	1080	Y	27	53	78	Undefined	Out Hospital
P18	8	17	1081	Y	19	41	14	DKA	Out Hospital
P19	14	24	1173	N	59	64	243	Hypoglycaemia	Out Hospital
P20	17	23	1237	N	76	70	222	CLD	Unknown
P21	8	8	1264	Y	24	20	49	DKA	Hospital
P22	19	21	1345	N	74	66	3	Undefined	Hospital
P23	17	30	1604	N	36	65	752	CKD	Hospital

DKA: Diabetic ketoacidosis, CLD: Chronic Liver Disease, CKD: Chronic Kidney Disease. The place of death was uncaptured in some records marked unknown.

**Figure 1:**
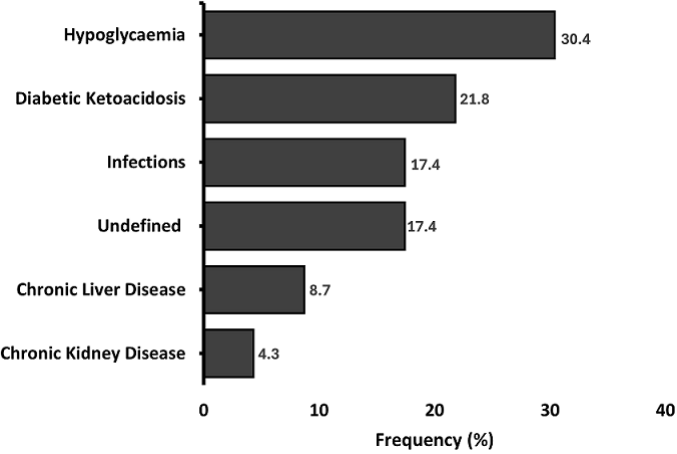
description of the causes of death

**Causes and circumstances of death:** the causes of death were hypoglycaemia (7 cases, 30.4%), diabetic ketoacidosis (DKA, 5 cases, 21.8%), infections (4 cases, 17.4%), undefined causes (4 cases, 17.4%), chronic liver disease (2 cases, 8.7%), chronic kidney disease (1 case, 4.3%), as shown in Figure 1. The majority of the death cases (17/23, 73.9%) had significantly low C-peptide levels (<200 pmol/L, severe insulin deficiency). Notably, 85.7% (6/7) of deaths due to hypoglycaemia had significantly low C-peptide levels (<200 pmol/L). Death occurred outside of the hospital in almost half (11/23, 47.8%) of the cases and 47.8% (11/23) of deaths occurred in participants who had been rolled out of the CDiC programme.

## Discussion

This study aimed to establish the number and causes of death amongst a group of CYP with diabetes recruited into the YODA study in Cameroon. We found that 53 months after the end of the YODA study recruitment, 23 death cases were recorded out of 313 participants enrolled in the cohort. The leading cause of death was hypoglycaemia, and the overall proportion with significantly low C-peptide levels (plasma C-peptide <200 pmol/L), a level where insulin treatment is absolutely needed for survival (severe insulin deficiency) [12], within this group was extremely high at 73.9%. Also, most of the hypoglycaemia-related death cases had extremely low C-peptide levels (plasma C-peptide <200 pmol/L). These results raise the question of whether, beyond the provision of free insulin treatment in resource-low settings, inherent biological factors such as endogenous insulin secretion, which can easily be estimated by measuring C-peptide levels, may be an important determinant of survival.

Mortality due to T1D in SSA has traditionally been associated with acute complications such as diabetic ketoacidosis (DKA) and hypoglycaemia, consistent with what we observed in this series. Several reasons have been advocated for high rates of DKA and hypoglycaemic-related deaths in T1D in SSA. Overall lack of awareness, late presentation at diagnosis, insufficient diabetes education, social deprivation, misuse of insulin treatment, and the absence of monitoring accessories are thought to contribute to the excess mortality seen in T1D in SSA [6,13]. Conversely, the causes of T1D-related mortality in high-income countries are predominantly chronic complications of cardiovascular and renal origins [14]. This disparity highlights the urgent need for improved healthcare infrastructure, access to medications, and education in low-resource settings, which will be important to prevent both acute and chronic complications of diabetes in CYP. Death occurred out of the hospital setting in almost half of the cases, which meant that the true cause of death may have been missed in some cases. This may explain why, in four cases, the cause of death was unknown. These further underscores the importance of awareness and that targeted diabetes-orientated education on sick-day attitudes, hypoglycaemia management and general health-seeking behaviours should be emphasised in this setting. We previously had shown that education and place of residence are independent contributors to T1D-related mortality in Cameroon [6]. The fact that almost half of the death cases had been rolled out of the insulin-provision initiative also provides an additional layer of complexity on the long-term sustainability of such initiatives within health systems in resource-limited settings and should be studied independently.

C-peptide, a peptide secreted in equimolar concentrations as insulin from the pancreas, has been demonstrated to be a reliable marker for endogenous insulin secretion in many clinical studies of people with diabetes [12]. Significantly low plasma C-peptide levels of <200 pmol/L, indicative of insulin deficiency, is one of the hallmark features of T1D and is recognised as a risk factor for hypoglycaemia [15]. We found that over three-quarters of all the death cases in this series and over 80% of hypoglycaemia-related deaths had significantly low C-peptide levels. This could be due to their reliance on exogenous insulin for survival, making them vulnerable to potential overdose, changes in insulin sensitivity, and other factors that can lead to low glycaemic levels [16]. These findings suggest that, beyond the provision and use of insulin in this SSA setting, endogenous insulin secretion may be a decisive factor in explaining mortality amongst CYP patients with T1D diabetes. This also highlights the importance of exploring the potential use of the C-peptide level as a prognostic marker for mortality risk in future research.

**Limitations:** this study has some limitations. The study's design is descriptive only, and therefore, we cannot ascertain causality between low plasma C-peptide and mortality within this cohort of children and young people living with type 1 diabetes. Also, death occurred outside of the hospital setting in almost half of all the reported death cases which could explain why the cause of death was unidentified in some cases. However, the findings from this study should drive further research on the association between endogenous insulin secretion and mortality or survival amongst children and young people living with T1D in Cameroon and in other sub-Saharan African countries.

## Conclusion

The majority of the reported death cases in this descriptive case series had significantly low plasma C-peptide levels, indicating severe insulin deficiency. This finding suggests that endogenous insulin secretion may be an independent contributor to mortality in children and young people living with type 1 diabetes in sub-Saharan Africa and must be considered and thoroughly investigated in future studies.

### What is known about this topic

- Historically, type 1 diabetes-associated mortality in Africa has been attributed to poor insulin access;
- Several free insulin provision programmes have been implemented in many African countries over the last two decades;
- Existing data shows mortality remains high in Africa compared to that seen in high-income settings despite free insulin programmes.

### What this study adds

- This study has been conducted in a well-phenotyped group of children and young people diagnosed with type 1 diabetes in an African setting;
- It describes the relationship between a biomarker (C-peptide) and the cause of death in children and young people with type 1 diabetes;
- This study proposes a hypothesis-generating idea to guide future research on the C-peptide and mortality in type 1 diabetes within African settings.
